# Supplementary material for: Stakeholders’ perceptions of the trends in contraceptive prevalence rate and total fertility rate in Ghana
Source: PLoS One. 2023 Jul 24;18(7):e0288924. doi: 10.1371/journal.pone.0288924 (PMC10365302; doi:10.1371/journal.pone.0288924)
Supplement: Attachment — Submitted filename: Response to reviwers comments.docx [file pone.0288924.s002.docx]

+233243335708

Email: gbagbofredyao2002@yahoo.co.uk or fygbagbo@uew.edu.gh

**25/04/2023**

To:

Bismark Dwumfour-Asare, MSc, PhD
Academic Editor
PLOS ONE

Dear Editor,

**Response to reviewers’ comments on paper entitled: " Qualitative Enquiry into Stakeholders’ Perceptions of the Trends in Contraceptive Prevalence Rate and Total Fertility Rate in Ghana through the lens of the Health Belief Model**

***[PONE-D-22-27258]***

I submit the response to the reviewers’ comments on the above research paper to be further considered for publication please.

Each comment has been carefully reviewed and a corresponding response given in yellow highlighted italics. The revised portions of the manuscript were also tracked using track changes for easy referencing.

Many thanks to the reviewers for the valuable comments to shape the paper.

Fred Y. Gbagbo, Ph.D.

Corresponding Author

**Edward K. Ameyaw**, Ph.D.

Co-Author

**RESPONSES TO THE ACADEMIC EDITOR’S COMMENTS**

1. Please ensure that your manuscript meets PLOS ONE's style requirements, including those for file naming.

**Response**

This has been ensured, thank you

2. During your revisions, please note that a simple title correction is required: Please remove the author names "1Fred Yao Gbagbo and 2Edward Kwabena Ameyaw" from the title on the online submission information.

**Response**

Noted and thank you.

3a. Please provide additional details regarding participant consent. In the ethics statement in the Methods and online submission information, please ensure that you have specified (1) whether consent was informed and (2) what type you obtained (for instance, written or verbal, and if verbal, how it was documented and witnessed). If your study included minors, state whether you obtained consent from parents or guardians. If the need for consent was waived by the ethics committee, please include this information.

**Response**

Thank you, we have provided these details in the ethics statement section.

3b. If you are reporting a retrospective study of medical records or archived samples, please ensure that you have discussed whether all data were fully anonymized before you accessed them and/or whether the IRB or ethics committee waived the requirement for informed consent. If patients provided informed written consent to have data from their medical records used in research, please include this information.

**Response**

We did not report a finding from a retrospective study of medical records or archived data.

4. Your ethics statement should only appear in the Methods section of your manuscript. If your ethics statement is written in any section besides the Methods, please delete it from any other section. Additional Editor Comments (if provided):
**Response**

Noted and done.

5. Our reviewers have done a thorough assessment of the manuscript and have given constructive comments and suggestions for the improvement of the manuscript to meet the standard required by the journal PLOS ONE before further consideration. The manuscript could have a potential for publication if the reviewers’ queries are addressed adequately moving forward.
**Response**

Thank you.

**RESPONSES TO THE REVIEWERS' COMMENTS:**

**Reviewer #1: GENERAL COMMENTS**
The authors did a good job. However, the paper talks about a concept that is already known, that is Contraceptive Prevalence Rate (CPR) and Total Fertility Rate (TFR) are inversely proportional; that is, an increase in CPR implies a decrease in TFR and vice versa. The paper also talks about another known fact which is a percentage increase in one does not always imply a reduction in the other by the same percentage. Based on the results obtained, the Health Belief Model may not have been the best approach. The points raised in the conclusion for future work is what the authors should have worked on. They have missed something crucial.

**Response**

We disagree with this comment. As clearly justified in our write up, the Health Belief Model is best fit for this study. We don’t think we missed anything crucial in this study to the best of our knowledge and expertise in this area of discipline. It will interest the reviewer to know that, this study was proceeded by a quantitative study looking at the associations between CPR and the TFR within 26 years (1988 to 2014 Ghana’s Demographic and Health Surveys) where to our surprise and contrary to previous public perceptions as being reiterated by reviewer here, we rather saw an inverse relationship/association between CPR & TFR in Ghana. It was based on the quantitative study that we decided to do the qualitative enquiry to find answers to our quantitative findings. We plan to do a systematic study looking at the various components and drivers of CPR and TFR in Ghana, hence the basis for our recommendation for further studies which we plan to do subject to availability of funding.

The authors said some restrictions exist in accessing data for the study. They did not state where data can be found.

**Response**

We did indicate in our write up that *‘data is available from the corresponding authors upon reasonable request’.* This is to ensure that we comply with one of the conditionalities by a research participant.

If the paper is to be published, it needs major revision.

**Response**

Noted and thank you for your inputs. We have revised it thoroughly.

**SPECIFIC COMMENTS**
1. The paper has a number of outstanding typographical/grammatical issues. A few examples are stated below:
i. Misplaced/wrongly used punctuation
a. Page 3 line2: “?” after service
b. Page 11: inverted comma to close quote by first participant is missing
c. Page12: inverted comma to close quote by participant is missing
d. Page 13: inverted comma to close quote by participant 10 is missing
e. Page 16: Quote by participants 5 & 7 is not in inverted commas
**Response**

Thank you, we have revised the grammatical/typographical errors.

ii. Both British and American English used
a. Page 6:
b. Page 14: … Family Planning Programs …
c. Page 14: quote by participant 14 … programme …
d. Page 9 line 6: ... familiarization
e. Page 9 line 9: … familiarization …

**Response**

Thank you, we have now followed British English throughout.

iii. Present and past tense have been mixed up in some instances
iv. Misnomers in some instances
a. “Tenant” instead of “tenet”
v. Abbreviations being used without stating it in full first

**Response**

Noted and thank you

a. page 7 line 5: what does the “DKT” stand for?

**Response**

DKT International was named in honor of **D.K.** **(Deep) Tyagi**. Mr. Tyagi was the assistant commissioner of family planning in charge of public motivation and education aspects of India's family planning program until he died of cancer in 1969 in New Delhi. We used DKT because that is the official name of the organization.

**REVIEWER #2:**

This important study attempts to provide an expert view of recent trends in CPR and TFR using qualitative research methodology. The study is timely and if published will provide relevant basis for sexual reproductive health interventions in Ghana and elsewhere. However, major issues have been identified and where possible suggestions to improve the quality of the manuscript have been provided to the authors.
**Response**

Noted and thank you

**BELOW ARE MY COMMENTS FOR THE AUTHORS.**
1. Theoretical underpinning
The health belief model is not an appropriate theory for this study since it focuses on individual level health behaviours. In addition, the findings do not emphasize the constructs of HBM. A relevant community/population level theory for this study could be “critical realism” – a theoretical framework that combines a realist ontology with an interpretivist epistemology. Thus, the view that unobservable structures cause observable events (e.g trends in CPR) and that these observations can be understood if we understand the mechanisms that cause them. The authors are at liberty to consider other appropriate theories.

**Response**

Noted and thank you. However, we think the tenets of the HBM provides us with the best explanation for our paper. Estimating CPR and TFR is usually first done at the individual level of health behaviors before a population estimation is done. We adopted the HBM because our paper focused more on estimates at the individual level (CPR &TFR) of which the aggregated effects became a key finding to be explored later in-depth where we will probably consider the theories being suggested. Additionally, the mechanisms that cause the trends in the CPR and TFR were not the core focus of this qualitative study.

2. Study design
(i) The authors report an integrated descriptive and exploratory research design with a qualitative approach. However, the element of integration of descriptive and exploratory designs is neither clear nor appropriate. This work is solely exploratory (qualitative) so should be reported as such.

**Response**

Noted and thank you

(ii) Many COREQ items have not been reported or are not clear from the reporting. Items 1-5, 7,8,13,23,27,28, and 32 seems unreported. When a reporting checklist (e.g. COREQ, SRQR, PRISMA etc..) has been used, it is always helpful to attach a list of all checklist items and where they have been reported in the manuscript.
**Response**

Noted and thank you. The COREQ checklist has been completed and attached

3. Data Analytical procedures
Since the framework analysis approach is a specialized form of thematic analysis, it should be sufficient for the authors to only report the framework analysis approach.

**Response**

Noted and thank you.

4. Results
(i) Contraceptive Prevalence and Total Fertility Ratio cannot be considered as themes since they are the main problems that were investigated. If the authors wish to consider them as themes, then they should be reported as themes developed in advance of data collection (deductive) and not as emerging from the data (inductive) [see COREQ item 26, derivation of themes].

Noted and thank you.

(ii) The authors have not provided a detailed description of the subthemes. What is seen is a presentation of several quotes with very scanty description of the findings that reflect authors’ synthesis of the data. For instance on “Barriers to contraceptive use”, the authors mention that “participants held a perception that there are various barriers to contraceptive use…”. However, they failed to detail what these main barriers were (as revealed through their analysis of the data). The authors have to develop minor themes for the barriers or state them in their findings before supporting them with quotes. Quotes are not to be used as substitutes for the research findings. Kindly note that this comment applies to all the themes reported in the findings and not just the barriers to contraceptive use.

Noted and thank you. detailed description of the subthemes have been provided


5. Discussion
Present a detailed explanation of your findings, clearly situating them in the context of what is already known. Authors’ should try to compare key finding to existing knowledge and provide explanation for findings and not merely restate them.
Response: We have provided detailed explanation of the findings and clearly situated them in the context of what is already known. Thus, we have compared our key findings with existing knowledge and provided explanations.
5. Other comments
Kindly proofread the manuscript to correct any grammatical errors.

**Response**

Noted and thank you

Reviewer #3: Thanks for the opportunity to review the manuscript titled: “Qualitative Enquiry into Stakeholders’ Perceptions of the Trends in Contraceptive Prevalence Rate and Total Fertility Rate in Ghana through the lens of the Health Belief Model”. The manuscript is of interest and falls within the scope of the journal. However, I have the following comments to strengthen the current version of the manuscript. Based on the comments that I have raised, I suggest a MAJOR REVISION.

**Response**

Noted and thank you

TITLE
1. “Qualitative Enquiry into Stakeholders’ Perceptions of the Trends in Contraceptive Prevalence Rate and Total Fertility Rate in Ghana through the lens of the Health Belief Model”. This title looks too long and will benefit from modifications. I proposed the following title: “Stakeholders’ Perceptions of the Trends in Contraceptive Prevalence Rate and Total Fertility Rate in Ghana”. In this way, you would still have the opportunity to comment on the health belief model in your work. Also, readers will have the opportunity to read the abstract so they would be able to know that this study is qualitative.
**Response**

Noted and thank you

ABSTRACT
Background
2. “Studies in Ghana have reported discrepancies between trends of Total Fertility Rate (TFR) and Contraceptive Prevalence Rate (CPR) as also reflected in the various Ghana Demographic and Health Survey (GDHS) reports”. You can modify this statement as: “Studies in Ghana have reported discrepancies between trends of Total Fertility Rate (TFR) and Contraceptive Prevalence Rate (CPR)”. After that, try to indicate the research gap calling for the study and the rationale of the study. Apart from that, in the background section, let it be clear why you employed the health belief model as a theoretical framework/construct for the study.

**Response**

Noted and thank you

Methods
3. You have to comment on the sample size and the sampling techniques in the methods section. In the methods section of the abstract, talk about how the health belief model influenced the design of your methods. I am sure a bit of information on these issues will do. It will also help readers to have a fair idea about how the health belief model was employed in the study.

**Response**

Noted and thank you

Results
4. Since you employed the health belief model, you have to organize your results based on the constructs of the health belief model. Per what you have currently done, it shows that you did not employ any model to drive your study. These issues should be fixed.

Thank you, we have done this.

Conclusion
5. In the conclusion section of the study, you have to tie the findings to the health belief model.

**Response**

Thank you, we have done this.

**MAIN WORK**
Introduction
6. Page 3, “CPR is commonly computed for married or in-union women between 15 and 49 years”. Are you able to explain further why this age range is mostly considered?

**Response**

Noted and done

7. Page 3, “It is a key indicator for measuring improvements in access to reproductive health service? I am not sure you need the question mark (?)…

**Response**

It was a typo error and has been deleted

Theoretical frameworks
8. Here, comment on some of the studies on contraceptives that have employed the health belief model. Also, talk about how you are going to employ the health belief model in your work.

**Response**

We have cited studies on contraceptives that have used the health belief model. We have also talked about how it was employed in our study.

9. “This model is a behaviour change framework that has been and is still widely used throughout the world as a theoretical model to guide health promotion programs. It is used to explain and predict individual changes in health behaviours. Based on our assumptions that contraception and fertility are informed by an individual’s reproductive intentions, the (HBM) aligns well with our study design as it defines the key factors that influence health behaviours as an individual's perceived threat to sickness or disease (perceived susceptibility), the belief of consequence (perceived severity), potential positive benefits of action (perceived benefits), perceived barriers to action, exposure to factors that prompt action (cues to action), and confidence in the ability to succeed (self-efficacy)”. These statements should be cited. Read about the following scholars and cite them in your study.

Rosenstock, I M. (1974a). Historical Origins of the Health Belief Model. Health Education Monographs Winter, 2(4), 328-335

Rosenstock, I M. (1974b). The Health Belief Model and Preventive Health Behavior. Health Education Monographs Winter, 2(4), 354-386

Rosenstock, I M. (1988). Enhancing Patient Compliance with Health Recommendations. Journal of Pediatric Healthcare, 2, 67-72.
**Response**

Noted and done, thank you.

Methods
Design
10. “We adopted an integrated descriptive and exploratory research design with a qualitative approach for data collection and analysis”. Are you able to expand this statement by justifying why descriptive and exploratory research design? This will give readers better perspectives on why descriptive and exploratory research design was used.

**Response**

Noted and done

Target institutions
11. “The main target population included officials of government and non-governmental organisations [NGOs] working in the area of Sexual and Reproductive Health in Ghana’. Apart from selecting the NGOs based on their area of focus, what other criteria did you employ? E.g. Based on the number of years that they have been into sexual and reproductive health and other issues? Please, do clarify as this may have impacted on their perceptions of contraception/sexual and reproductive health ….

**Response**

To us the participants' perceptions of CPR and TFR were valid despite the duration of exposure/involvement in contraception/sexual and reproductive health issues in Ghana and internationally.

12. “Marie Stopes Ghana, Planned Parenthood Association of Ghana (PPAG), DKT International Ghana, Ghana Health Service, Total Family Health Organisation (TFHO), United Nations Population Fund (UNFPA) and Alliance for Reproductive Health Rights (ARHR)”. How many years have these NGOS being in operation?

**Response**

To us the participants' perceptions of CPR and TFR were valid despite the duration of exposure/involvement in contraception/sexual and reproductive health issues in Ghana and internationally.

13. “Heads of public institutions or the Focal persons on population and related issues in these institutions were also selected to participate in the study: National Population Council (NPC) Ghana, the Ghana Health Service (GHS), The Ministry of Health (MoH), The Ministry of Women, Gender and Social Protection (MoWGSP)”. How many years have they been working with these institutions? Any information on that? This information could influence the quality of the information these stakeholders will provide.

**Response**

To us the participants' perceptions of CPR and TFR were valid despite the duration of exposure/involvement in contraception/sexual and reproductive health issues in Ghana and internationally.

14. “Besides, some academics with demonstrable evidence of working in the area of sexual and reproductive health were targeted from the following institutions: University of Ghana (specifically 8 Regional Institute of Population Studies [RIPS] and the Department of Population, Family and Reproductive Health), University of Cape Coast (thus Department of Population and Health”. How many years have they been in academia?

**Response**

To us the participants' perceptions of CPR and TFR were valid despite the duration of exposure/involvement in contraception/sexual and reproductive health issues in Ghana and internationally.

Participants selection
15. “As the emphasis of qualitative research is not on large sample sizes, recruitment of participants was done until a point of saturation was reached on major issues that were outlined to be examined”. I don’t seem to agree with this statement because you already have a pre-determined categories of people you were going to gather data from per what you have highlighted in your target institutions. Hence, you need to reconsider your reason. I however, agree with you on the basis that a sample size of 12 is good enough for a qualitative study since the emphasis is not on generalization.

**Response**

Noted and thanks

16. “In all, 15 stakeholders consented and participated in the study”. Did they consent orally or written and why? Such information may help readers…

**Response**

Noted and thank you. This has been clarified under ethical issues

17. “Source: Authors’ Construct 2022”. Remove this information, you don’t need it especially in publications.

**Response**

Noted and thanks

Data Collection Procedure
18. Were interviews conducted in English? if yes, why? What were some of the trustworthiness/rigor issues such as credibility, transparency, dependability, and confirmability that were considered in your study? Include information on how you applied these issues in your work

Noted and thanks

. One key issue is that you are yet to capture information on how you design your data collection instrument to reflect the domains/dimensions of the health belief model… So far, the above information is missing in your work

Noted and thanks

Further, as part of the data collection procedure, you should also be able to provide information on research team and reflexivity. This will also include your positionality and intersectionality issues. All these information can be captured as part of the research team and reflexivity…

Noted and thanks

What is your position in this study? Are you entering this research as an outsider/insider or what? How does your outsider/insider perspective shape the outcome of your study or influenced how you interacted with the participants during the field work?

Noted and thanks

19. “Detailed field notes were taken by a dedicated note taker during interviews”. But I guess you said it was done via phone, right? If that is the case, did you put it on loudspeaker (because if it was not on loudspeaker, your note taker cannot hear the responses of the participants)? If yes, then you should explain how you did not breach the issue of confidentiality and privacy? Also, did you allow the note taker to sign any confidentiality form? If yes, you need to include this information in the work? If no, why? Did you give training to the note taker? What was the training about? How many days for the training? These issues should be commented in the work.

**Response**

The initial contacts were done on phone but the actual interviews were done one on one by face-to face for the same reasons the reviewer raised.

20. “Data collection lasted for two months”. Just stating data collection lasted for two months may not be enough, just be specific? For instance, January 2022-March 2022. This will make things clearer for everyone who is reading the work.
**Response**

Data collection took place between July to August 2022.

Data Analytical procedure
21. “With these approaches, all audio-recorded interviews were first transcribed verbatim in the original interview language (English)”. This contradicts with your earlier statement that: “ Detailed field notes were taken by a dedicated note taker during interviews”. Please, recheck…
**Response**

Noted and revised to reflect what we did during the field work

Results
22. Terrific! This was what I was talking about in the methods section under target institutions. I mean the years of experience in family planning. You can add it now…

**Response**

This was a key finding. We could not have had this information prior to the field work. Although we reported on it at this stage, it really did not matter in the qualitative analysis since we did not adopt any quantitative statistical analysis to see the impact of the years of experience/exposure to SRHR on responses given.

23. “Table 2: Socio-demographic characteristics of participants”, just providing information on the years of experience in family planning alone cannot constitute socio-demographic information of the participants. Do you have data on their gender, age, education level etc.? If yes, add them especially the gender?

**Response**

Noted and thanks

24. I suggest you present the results based on the domains of the health belief model. Also, the authors have provided quotations without any detailed write-up. The quotations should be well explained.

**Response**

Noted and thanks

Discussion
25. The discussion is quite okay. The authors should well connect their findings to the health belief model. Also, the authors should be strengthened the implication part. For this reason, a section focusing on policy, practice and research implications should be devoted for that.

**Response**

Noted and thanks
