# Supplementary material for: Stakeholders’ perceptions of the trends in contraceptive prevalence rate and total fertility rate in Ghana
Source: PLoS One. 2023 Jul 24;18(7):e0288924. doi: 10.1371/journal.pone.0288924 (PMC10365302; doi:10.1371/journal.pone.0288924)
Supplement: Attachment — Submitted filename: Response to reviwers comments Rev 2.docx [file pone.0288924.s004.docx]

+233243335708

Email: gbagbofredyao2002@yahoo.co.uk or fygbagbo@uew.edu.gh

**09/06/2023**

To:

Bismark Dwumfour-Asare, MSc, PhD
Academic Editor
PLOS ONE

Dear Editor,

**Response to reviewers’ comments on paper entitled: " Qualitative Enquiry into Stakeholders’ Perceptions of the Trends in Contraceptive Prevalence Rate and Total Fertility Rate in Ghana through the lens of the Health Belief Model. [PONE-D-22-27258R2]**

I submit the response to the reviewers’ comments on the above research paper to be further considered for publication please.

Each comment has been carefully reviewed and a corresponding response given in yellow highlighted italics. The revised portions of the manuscript were also tracked using track changes for easy referencing.

Many thanks to the reviewers for the valuable comments to shape the paper.

Fred Y. Gbagbo, Ph.D.

Corresponding Author

**Edward K. Ameyaw**, Ph.D.

Co-Author

**RESPONSES TO THE ACADEMIC EDITOR’S AND REVIEWERS’ COMMENTS**

1. To a large extent, you have addressed the issues I raised earlier. However, one outstanding issue is the concern about the appropriateness of your theoretical framework to which you provided the quoted response below:

Noted and thank you. However, we think the tenets of the HBM provides us with the best explanation for our paper. Estimating CPR and TFR is usually first done at the individual level of health behaviors before a population estimation is done. We adopted the HBM because our paper focused more on estimates at the individual level (CPR &TFR) of which the aggregated effects became a key finding to be explored later in-depth where we will probably consider the theories being suggested. Additionally, the mechanisms that cause the trends in the CPR and TFR were not the core focus of this qualitative study.

A few issues in your response need considering and I think critically considering your theory will greatly improve your manuscript:

a) CPR & TFR are not estimated or cannot be estimated at the individual level since they are population health indicators. Rates are measures of the frequency with which an event occurs in a defined ‘***population’*** over a specified period of time and are not individual health measures.
b) In the last statement of your response, you make the claim that your study did not focus on examining the mechanisms that cause the trends in CPR & TFR. I do not think that is accurate since your *main objective of the study was to examine the various stakeholders’ perceptions of the trends of CPR and TFR in Ghana*. You sought their perceptions on *why* (the mechanisms or underlining reasons) for the observed trends. If this was not what you sought to do, then it is not clear at this stage what you intended to do.

2. In your introduction you make the following claim: *We employed the Health Belief Model as a theoretical framework/construct for the study to ‘****explain’*** *and ‘****predict’*** *individual changes in health behaviours resulting in trends in CPR and TFR from stakeholders’ perspectives.*

This was not done anywhere in your manuscript. I once again suggest that your critically consider changing your theoretical framework. If you used the HBM then it is expected that at the minimum, your results section is organized to contain the constructs of the theory. So far, this is not done. There is no theme/data on five of the six constructs of HBM in your results; perceived severity, perceived susceptibility, perceived benefits, ‘*perceived barriers’*, cues to action, and self-efficacy. The only construct you considered is perceived barriers. To be honest with you, I do not see how you could justify your reliance on HBM for this study because clearly your data is not explained by the constructs of the theory.

**Response**

*After careful reflections on the reviewer’s comments on the suitability of the Health Belief Model used earlier and through review of the literature, we have revised our paper to focus on*

*The Theory of Planned Behavior (TPB). This Theory suggests that individuals act rationally according to their attitudes, subjective norms, and perceived behavioral control (*[*Ajzen, 1985*](https://www.sciencedirect.com/science/article/abs/pii/074959789190020T?via%3Dihub#BIB4)*;* [*Ajzen, 1987*](https://www.sciencedirect.com/science/article/abs/pii/074959789190020T?via%3Dihub#BIB5)*; Ajzen, 1991). In this regard, to use contraceptive(s) to prevent pregnancy and childbirth, an individual must evaluate it positively, believe it is worthwhile, and perceive it to be within their own control. Originally, the theory helps to explain attitudes of individuals towards a behavioural change or modification. However, in the present study, it is aiding us to engage stakeholders to offer explanation to the attitude of Ghanaian women toward contraception and how this has shaped and continues to shape the country’s TFR. The use of contraceptives involves a number of key factors, including attitude, subjective norm, and perceived behavior control. Subjective norm is based on the social context, attitude is based on results, and perceived behavior control is based on challenge. The person's attitude, subjective norms, and sense of control have an impact on their decision to use contraceptives to regulate their fertility. Stakeholders in healthcare have a vested interest in contraceptive decisions. Hence, through the lens of the TPB, we are able to explore the stakeholders’ expert opinions about contraceptive use and fertility trend in Ghana, to guide policy and fertility regulation implementation strategies. Using the TPB to explain Stakeholders’ Perceptions of the Trends in Contraceptive Prevalence Rate and Total Fertility Rate in Ghana is strongly supported by our empirical data, which suggest that attitudes, subjective norms, and perceived behavioral control are critical contributory factors towards fertility regulation (Ajzen,and Klobas, 2013).*

**References**

Ajzen, I., 1985. *From intentions to actions: A theory of planned behavior* (pp. 11-39). Springer Berlin Heidelberg.

Ajzen, I., 1987. Attitudes, traits, and actions: Dispositional prediction of behavior in personality and social psychology. In *Advances in experimental social psychology* (Vol. 20, pp. 1-63). Academic Press.

Ajzen, I., 1991. The theory of planned behavior. *Organizational behavior and human decision processes*, *50*(2), pp.179-211.

Ajzen, I. and Klobas, J., 2013. Fertility intentions: An approach based on the theory of planned behavior. *Demographic research*, *29*, pp.203-232.

Thank you
